# Computer-tailored physical activity behavior change interventions targeting adults: a systematic review

**DOI:** 10.1186/1479-5868-6-30

**Published:** 2009-06-03

**Authors:** Leonie Michelle Neville, Blythe O'Hara, Andrew Milat

**Affiliations:** 1 Centre for Health Advancement, New South Wales Department of Health, Locked Mail Bag 961, North Sydney NSW 2059, Australia

## Abstract

**Background:**

Increasing physical activity is important in the promotion of better health. Computer-tailored behavior change programs have shown promise in changing lifestyle risk factors.

**Purpose:**

To provide a narrative systematic review describing the range of evidence on 'second' and 'third' generation computer-tailored primary prevention interventions for physical activity, to determine their effectiveness and key characteristics of success. Unlike previous reviews, this review used specific criteria to measure the external validity of studies, was exclusive to primary prevention interventions in which tailoring was generated through an expert system, and excluded first generation computer-tailored interventions.

**Methods:**

Computer-tailored intervention studies published from January 1996–2008 were identified through a search of five databases: Medline; Embase; PsycINFO; CINAHL; and All EBM Reviews and by examining reference lists of relevant articles.

**Results:**

Seventeen articles were included, describing the evaluation of 16 interventions, ten of which found significant positive effects of the computer-tailored interventions on physical activity or weight reduction outcomes.

**Conclusion:**

The evidence of effectiveness for computer-tailored physical activity interventions is inconclusive. They have potential to reach large groups of people however there is uncertainty whether reported effects are generalizable and sustained.

## Introduction

At least 60% of the world's population does not meet the recommended levels of physical activity required to induce health benefits [1]. Levels of inactivity are high in nearly all developed and developing countries, with more than half the adults in developed countries insufficiently active [2]. This is certainly the case in Australia, where in 2004 about 50% of the adult population were insufficiently physically active [3]. This is a significant problem as physical inactivity is estimated to cause 1.9 million deaths with a burden of 19 million disability-adjusted life years globally, significantly contributing to many cancers, diabetes and heart disease [4].

There is growing evidence that behavior change programs using computer-tailoring can be effective in changing lifestyle risk factors [5]. Computer-tailoring refers to the documentation of participant information using a computerized expert system, which then generates feedback and advice based on such information. Computer-tailored interventions have been classified into three generations, according to their mode of delivery. First generation interventions are delivered through printed materials such as letters, reports and pamphlets. Second generation interventions are delivered through interactive technology or desktop applications such as websites, email and CD-ROM programs [6,7]. Third generation interventions are relatively new and include mobile and remote devices such as mobile phones and handheld computers which may enhance the potential for timely feedback and assessment [6].

Computer-tailoring is promising as a strategy for health education [5]. Firstly, like personal counseling, participants' behaviors are assessed and the results then used to generate individualized feedback and advice [7], making the health information received personalized and provided at a relatively lower cost than interpersonal counseling [5]. Participant's behaviors can be compared with current recommendations, the behaviors of peers and previous assessments [8]. Feedback can then be provided that is relevant to performance levels, awareness, motivation, self-efficacy, expectations and goals [5]. Secondly, computer tailoring has potential for wide distribution due to its application to electronic non-print media such as the Internet, providing an opportunity for remote access to the intervention [5].

The limitations of computer-tailored interventions are that the participant must answer many questions to be assessed accurately, allowing for the provision of reliable and individualized advice [9]. Feedback is based on the participant's self-reported behavior, which may result in incorrect estimates of behavior and mismatched feedback and advice [10]. These limitations may be minimized through the use of a combination of validated self-reports with more objective measures of physical activity behavior [5].

Previous well-conducted systematic reviews on computer-tailored interventions [5], web-based interventions [11,12] and interventions using interactive technology [6] targeting physical activity and dietary behaviors indicated that further research was required to form any conclusions on the effectiveness of such interventions in the promotion of physical activity or healthy weight, but the evidence was promising. We investigated the potential of developing computer-tailored physical activity behavior change interventions targeting Australian adults. The abovementioned reviews had significant relevance and application to the development of such strategies, however had a different purpose, focus and inclusion criteria and considerable time has lapsed since their publication in this rapidly developing field [5,6,11,12].

These reviews noted the significant heterogeneity of such studies. When there is significant heterogeneity of studies it is considered more appropriate to undertake a narrative systematic review than a meta-analysis and to describe the variation in findings rather than attempt to combine findings into one overall estimate of effect [13].

The aim of this study was to conduct a narrative systematic review which would describe the range and quality of available evidence on second and third generation computer-tailored primary prevention interventions for physical activity behavior change and to determine their effectiveness and key success factors.

## Methods

### Data Sources and Search Strategy

Literature searches were conducted to retrieve articles from January 1996 to January 2008 that were written in English using five databases in February 2008: Medline, Embase, PsycINFO, CINAHL and All EBM Reviews. Previous systematic reviews on computer-tailored physical activity interventions did not identify any studies prior to 1996 [5,6]. The search consisted of a combination of each of the following terms to represent computer-tailored or expert systems: expert system; or web based; or computer tailor*; or computer based; or Internet based; with each of the target domains: physical activity; overweight; obesity; and weight loss. Additional articles of relevance were sought by reviewing the reference lists of included articles and previous systematic reviews [5,6,11,12,14-16].

### Selection criteria

Articles identified through the literature search were restricted to those written in English and published in a scientific journal between January 1996 to January 2008, inclusive. Only randomized controlled trials (RCTs) or quasi-experimental designs with pretest and post test behavioral outcome data were included.

For inclusion in this review articles had to describe the evaluation of a 'second' or 'third' generation computerized intervention in which tailored physical activity advice was generated through a computerized system and delivery was inclusive of but not exclusive to the electronic technology. Such interventions were considered tailored if the advice or feedback provided was specific to the individual and based on an individual assessment and their characteristics. Further, the intervention had to target adults for physical activity as a primary prevention strategy. The review used the same definition of primary prevention as Kroeze et al [[5]:206], namely "...the initiation of lifestyle or behavioral changes to prevent the onset of chronic diseases in apparently healthy participants." Physical activity behavior or fitness levels had to be described as the primary outcome measure.

Studies were excluded that had an intervention with significant face-to-face contact involving counseling in the main treatment arm. Interventions with limited interpersonal contact such as: provision of computer-tailored feedback through telephone or email; initial one-off face-to-face sessions for the purpose of instructing participants in the use of the technology or data collection (not for the purposes of behavioral counseling) were included. Studies that had additional treatment arms such as face-to-face sessions were included, however the effects reported in this review are only that of the treatment arm with none or limited interpersonal contact as described above.

Articles were also excluded if they met the following criteria:

• Conference abstracts, dissertations, commentaries, descriptions of the technology or information architecture, description of intervention development only

• The target group for the intervention was caregivers, health professionals or those with a manifested chronic disease state and/or recruitment occurred using chronic disease registries

• Intervention described was a maintenance strategy for a previous intervention that had not been generated through such a system.

Where studies addressed multiple behaviors, only physical activity and change in body mass or weight outcomes were considered. Although not the main purpose of this review, when physical activity behavioral effects were absent or conflicting, physical activity mediator outcome measures were considered.

### Data synthesis

The Australian National Public Health Partnership (NPHP) guidelines for evaluating evidence on public health interventions [17] and previously published reviews [5,6,11] were used as a guide to reviewing and summarizing the studies included. Each article was reviewed by two of the authors with the following information extracted and tabulated: Intervention context: setting, target population, recruitment methods, eligibility/inclusion criteria, exclusion criteria, sample characteristics, incentives offered; Intervention: description of treatment groups & control, delivery process, intensity, duration, use of a behavior change theory, the communication channel/delivery mode; Study design and evaluation: study design, randomization, methods, analysis, length of follow-up; Outcome measures: primary & secondary, instruments used, validation; Findings: generalizability, sustainability, retention; Strengths & limitations of the study. These two authors independently performed a quality coding assessment of all studies, which consisted of eighteen criteria symbolizing the quality of the intervention and the study internal and external validity (Table 1). These characteristics were adapted from those used in previous reviews [6,12], the Australian NPHP guidelines for evaluating evidence on public health interventions [17] and external validity criteria outlined by Glasgow & Emmons [18]. Each criteria had the same value or weighting, the sum of which was used as a validity score, calculated as a percent of the maximum obtainable score. Ranking disagreements were discussed by all authors until consensus was reached. All authors reviewed both the summarized review of studies and the quality assessment then convened to reach consensus on the strength of evidence.

**Table 1:** Study internal and external validity coding criteria

	**Criteria description**	**Scoring for criteria**
**Internal validity criteria**		

1	Was the method of randomization appropriate?	Y = 1; N = 0

2	Were baseline groups equivalent on important demographic measures?	Y = 1; N = 0; UK = 0
	
	If No, was analysis conducted to estimate/adjust for effect of demographic measure on outcomes?	Y = 1; N = 0; UK = 0

3	Did the design of the study include comparison to a no treatment control group or a group with either no technology or no tailoring?	Y = 1; N = 0

4	Was retention rate ≥ 80% at post-test/post-intervention follow-up?	Y = 1; N = 0

5	Were Outcome Measurement instruments valid? Was there a description of instrument reliability/validity (reference or coefficients) OR did they use a well-established known valid measure?	Y = 1; N = 0

6	Was an objective measure of behavior change used or did they rely solely on self-report measures?	Objective = 1; Self-report only = 0

7	Was power analysis reported to determine sample size?	Y = 1; N = 0

8	Were analyses conducted with consideration for missing data that maintain fidelity of the randomization (e.g. intention to treat, imputation)? Note: if 100% retention then N/A	Y = 1; N = 0

9	Was the intervention based on theory?	Y = 1; N = 0

**External validity criteria**		

1	Were recruitment methods and/or inclusion & exclusion criteria sufficiently described?	Both = 1, Either = 0.5; None = 0

2	Were participation/recruitment rates provided OR Are analyses reported on the similarity and differences between participants versus either those who decline or the intended target audience (individuals or settings)?	Y = 1; N = 0

3	Was a large heterogeneous sample used? Was the representativeness of participants described? Was a homogenous/heterogeneous sample sought for target population? Do the exclusion criteria used reduce the generalizability of findings?	Generalizable population = 1

4	Was the representativeness of the setting described? Was the study conducted in an uncontrolled/controlled setting? Can their findings only be generalized to the limited conditions within which the research was carried out?	Generalizable setting (real-life) = 1; Controlled = 0

5	Were all participants who entered trial accounted for at its conclusion i.e. Are data on attrition by condition reported OR was dropout rate described?	Y = 0.5; N = 0
	Are drop-outs' compared to completers OR are the dropout's characteristics and reasons for drop-out described?	Y = 0.5; N = 0

6	Was the use of comparison conditions relevant to real-world decisions? (the computer-tailored treatment group was compared with either non-technology or non-tailored or alternative programs rather than no treatment)	Y = 1; N = 0

7	Are data on the costs presented?	Y = 1; N = 0

8	Was there sufficient description of the intervention, including: method of tailoring, duration and intensity (amount of contact time required)?	Y = 1; N = 0

9	Are data reported on maintenance or longer-term effects?	Short-term = 0; Medium term = 0.5; Long-term = 1

## Results

### Study selection

The initial cross-database literature search yielded 769 publications. After removing duplicates and reviewing the title and abstract of these publications against the inclusion criteria the number of eligible published articles was 26. After reviewing the full articles, 14 were excluded for meeting one of the exclusion criteria, leaving 12 articles. The search of reference lists of relevant publications yielded three articles. Two additional articles were included: one was identified in the same journal issue of an included study and one was in press, which was identified by a colleague.

A total of sixteen interventions, evaluated in seventeen separate studies aimed to increase physical activity (Additional file 1) [19-35]. Two articles described the post-test [27] and long-term follow-up [26] of the one intervention program. Another two articles were related, but described an adapted version of the intervention and its trial in different settings [23,24]. One article described the physical activity component only [35] of a two armed RCT of parallel nutrition [36] and physical activity interventions.

Six articles described the evaluation of five computer-tailored multi-component health interventions that targeted both physical activity and dietary behaviors [19,20,26-28,30]. Four studies also measured weight reduction outcomes [19,20,28,33].

### Outcome effects

Thirteen studies reported short to medium-term positive effects on physical activity, ranging from ten weeks to six months post-test and from two weeks to five months post-intervention [19-25,27,30-32,34,35] and one reported positive effects at long-term post-intervention follow-up (Table 2) [26]. Of these 14 studies: seven reported a significant short to medium-term effect in favor of the computer-tailored intervention over a control group; [21,22,24,27,30,32,35]; and seven reported a significant positive effect on physical activity over time for both the computer-tailored intervention and a comparison treatment group but no significant between group effects, for one of which the effect was only found for those inactive at baseline [31]. The comparison treatment groups for these studies are outlined in Table 2. Of the three studies which did not find positive effects on physical activity behavior outcomes, two reported positive effects on physical activity mediators such as self-efficacy and physical activity self-regulation strategies [28,29] and two reported significant positive effects on weight reduction outcomes [28,33].

**Table 2:** Outcome effects* and validity scores of reviewed studies

**Study**	**Dietary Behavior**	**Physical activity**	**Weight reduction**	**Internal validity (%)**	**External validity (%)**
**Physical activity**					

Hurling (2007) [21]		+^a^		89	44

Marcus (2007) [22]		+^b ^(6 months only, not maintained at 12 months)		89	56

Spittaels (2007b) [24]		+^b^		56	78

Steele (2007) [25]		(+)^a^		89	44

Marshall (2003) [31]		(+)^c(sub) ^(for those not active at baseline only)		78	67

Napolitano (2003) [32]		+^b^		67	44

Hager (2002) [34]		(+)^d^+^e(sub) ^(for males only)		56	39

Pinto (2002) [35]		+^f ^(3 months only, not maintained at 6 months)		67	67

**Combined physical activity, dietary behavior &/or weight reduction**					

Booth (2008) [19]	(+) fat only^f^	(+)^f^	(+)^f^	56	67

Cook (2007) [20]	(+)^g ^(overall diet including fat)	(+)^g^	(+)^g^	67	33

Spittaels (2007a) [23]		(+)^dh^	(+)^dh ^& +^dh(sub)^	78	72

Vandelanotte (2005 & 2007) (6 month post-test) [27]	+ fat^b^	+^b^		44	56

(2 yr follow-up) [26]	(+) fat^h^	(+)^h^			

Winett (2007) [28]	+ fibre, F&V^b^	M^b^	+^b ^(3 months only, not maintained at 6 months)	89	61

Hageman (2005) [29]		M-^d^	+/-^d^	78	56

Kypri (2005) [30]	+ F&V^b^	+^b^		56	39

Veverka (2003) [33]		ND	+^b^	78	39

Key:

+ significant difference over time between treatment & control group OR significant difference between groups at post-test

(+) significant difference within groups over time but no significant difference between groups

+/- conflicting results

M positive effects on behavior mediators but not on actual behavior

M – negative effects on behavior mediators

(sub) outcome effect only for sub sample (1 worksite, n = 57)

ND no difference

*Outcome effects are reported for the effect of the computer-tailored intervention group as compared to the following control groups:

a Tailored (personal) verbal

b No treatment control group

c Tailored print comparison group

d Generic computerized comparison group

e Computerized comparison group with different method of tailoring

f Comparison treatment group on different/additional targeted behavior

g Generic print comparison group

h Comparison group of lower intensity

Two of the studies mentioned above with short to medium-term positive effects on physical activity were not able to report positive effects at longer-term post-test follow-up [22,35]. It is worth noting the positive medium-term effects on physical activity found for one intervention at post-test [27] were confirmed at long-term post-intervention follow-up, however there was no control group and a potential drop-out bias at the long-term follow-up [26]. The authors of this study noted the limitations of these findings in terms of real-life effectiveness, generalization and application to practice due to the controlled setting and motivated sample [26,27]. However medium-term post-test positive effects on physical activity were also found for an adapted version of this same intervention program implemented in a real-life setting using the Internet [24].

### Study quality: Internal & external validity characteristics

#### Internal validity scores

The internal validity scores ranged from 44–89%, with an average of 71% for all studies and 67% for those studies reporting significant positive between group effects on physical activity outcomes (Table 2). Of the eight studies which had an above average internal validity quality rating (> 71%) five found positive between-group effects: two for physical activity outcomes and three for weight reduction outcomes. Most quality criteria reflecting the internal validity of studies were met by a majority of studies, with the exception of three quality criteria: reporting a rationale for sample size; the use of objective measures of behavior change; and conducting analyses with consideration for missing data that maintained the fidelity of the randomization. Low internal validity scores reported by studies reporting significant positive between group effects on physical activity outcomes were mainly due to reliance on self-report measures of behavior, poor retention rates and no description of the following: whether baseline groups were demographically equivalent; and sample size determination.

#### External validity scores

The external validity scores ranged from 33–78%, with an average of 54% for all studies and 52% for those studies reporting significant positive between group effects on physical activity outcomes (Table 2). Of the nine studies which had an above average external validity quality rating (> 54%) six reported positive between-group effects: four for physical activity outcomes and two for weight reduction outcomes. Only a minority of studies met the following external validity criteria: reporting the maintenance of long-term post-intervention effects; reporting on intervention costs; using representative samples; and reporting on participation or recruitment rates or the similarity and differences between participants to either those who declined participation or the intended target audience.

### Intervention and study characteristics

#### Mode of delivery

All interventions were classified as 'second' generation computerized interventions with only one including additional mobile phone technology, considered as 'third' generation technology [21]. The majority of interventions were delivered using the Internet and/or email [19-21,23-25,28,29,31-34], followed by desktop computer programs [27,30] and telephone [22,35].

Of 11 studies that isolated the effect of the technology by comparing the computer-tailored intervention group to either a no-treatment or waiting list control, or a comparison treatment group receiving print materials or personal counseling, nine found significant between group effects: 6 for physical activity [21,22,24,27,30,32] and three for weight reduction outcomes [23,28,32]. Of these nine studies, six were delivered using the Internet and/or email [21,23,24,28,32,33], two were delivered using desktop computer programs [27,30] and one was delivered by telephone [22]. One Internet-based study also used mobile telephone technology [21].

#### Study sample

Baseline sample size ranged from 31 to 1071. Nine studies either described dropouts compared to study completers and/or described reasons for dropout [19,22-24,26,28,30,32,35], five studies reported a rationale for sample size [25,28-31], and only four studies reported on the characteristics of participants compared to the target population [23,24,28,31].

The generalizability of findings was a limitation of all studies due to one or more of the following reasons: a small or unrepresentative sample; an unrepresentative target population, or the controlled setting within which the study was conducted. The majority of study samples usually consisted of healthy adults recruited through community settings, [19,21,22,24-29] followed by the workplace, [20,23,31,32,34] primary care [30,35] and one in the military [33]. The majority of interventions recruited self-select volunteer individuals [19-25,27-30,32-35]. Many studies used additional eligibility or exclusion criteria related to medical conditions [22-24,27,28,32,33,35] age, [21,23,24,27,29,33,35] health behavior status [21,22,25,29,32,35], medication [19,21,22,33] body mass index (BMI) [19,21,22] and gender [29].

The majority of samples were predominately female, well educated and Caucasian. Of the 17 studies, 14 reported a predominately female baseline or follow-up sample [19-22,24-29,31,32,34,35] with a median proportion of 67% for all studies. Eleven of the 13 studies reporting on education level of their baseline or follow-up sample had a predominately well-educated sample, as determined by level of educational attainment or years of education, [19,20,22-24,26,27,29-32,34] with a median proportion of 71% for all 13 studies. All seven studies reporting on the ethnic-racial background of their baseline or follow-up sample had a predominately Caucasian/White sample, with a median proportion of 90% [19,20,22,29,32,34,35].

Of the seven computer-tailored interventions reporting significant positive between group effects on physical activity outcomes [21,22,24,27,30,32,35] all but one study [30] had a predominately female baseline or follow-up sample and five reported a predominately well-educated sample [22,24,27,30,32].

#### Duration and exposure

Of the seven computer-tailored interventions reporting significant positive between group effects on physical activity outcomes [21,22,24,27,30,32,35] five were multiple exposure interventions, ranging from nine weeks to 12 months duration [21,22,24,32,35] three of which ensured weekly exposure at minimum [21,32,35] and the remaining two approximately monthly [22,24]. Two of the interventions involved a single exposure for the participant to a computer-tailored program [27,30] one of which was followed up at two years [26].

Napolitano et al [32] questioned whether participants received adequate exposure to their three month intervention after the first month as most of the physical activity outcome changes that occurred did so in the first month and they received anecdotal feedback from participants suggesting that changing the static website over time would be worthwhile. Steele et al [25] reported sustained physical activity outcomes at five months and noted their content was delivered on a weekly basis to enhance user engagement over time.

#### Intensity

Four interventions compared computer-tailored intervention groups which differed in intensity [19,23,24,27] only one of which found significant differences between the groups, reporting the higher intensity intervention group had greater improvements than the lower intensity group, but only for a sub sample of participants [23].

Two web-based studies compared computer-tailored intervention groups with the same intervention in addition to personal support [25,28]. One study found that the intervention group receiving the additional social supports had better outcomes, however both groups had significantly better outcomes than a waitlist control group [28]. The other study found no enhanced physical activity outcomes for additional personal contact, concluding that the Internet intervention was as effective as the face-to-face intervention [25].

#### Use of theory

Of the seven computer-tailored interventions reporting significant positive between group effects on physical activity outcomes [21,22,24,27,30,32,35] a wide range of theories were used, most commonly the transtheoretical model [22,24,27,32,35], social cognitive theory [22,32,35], and the theory of planned behavior [24,27]. These were also the most commonly used theories overall. Other theories of successful interventions included decision making theory [35] and social psychological theories (social comparison, decisional balance, elaboration likelihood, Goal [21].

#### Tailoring

Only two studies isolated the effect of the tailoring by comparing the computer-tailored intervention group to a comparison treatment group receiving generic information via the same technology. Neither of these studies reported significant between-group differences in physical activity outcomes [23,29].

The most commonly used methods of tailoring were providing feedback tailored to the participant's motivational stage of change [20,22-24,27,31-35] followed by providing feedback that compared participant's behavior to current recommendations [19-21,23,24,27,28,30,35]. The majority of studies tailored feedback in more than one way [19-21,23,24,27,28,30,35], however the combination of tailoring varied. Other ways of tailoring included providing feedback that compared the participant's behavior to previously set goals [19,21,28,29], the behavior of peers [21,30], participant's previous behavior, [29] and feedback tailored to participant's self-efficacy [23,24,27,29] their intentions and attitudes, [23,24,27] and their perceived benefits and/or barriers to behavior [21,29].

#### Outcomes and instrument validity

Only one physical activity study did not indicate the use of valid instruments to measure behavioral outcomes [30]. The most commonly used instrument for measuring physical activity outcomes was the long or short version of the International Physical Activity Questionnaire [21,23,24,26,27,31]. Other questionnaires included the Seven-day Physical Activity Recall [22,34,35], the Physical Activity Readiness Questionnaire [28,32], the Active Australia questionnaire [19,25], the Modified Seven-day Activity Recall [29], the Behavioral Risk Factor Surveillance System [32] and the Veterans Specific Activity Questionnaire [28].

Objective physical activity outcome measurement instruments included the pedometer [19,28], accelerometer [21], functional capacity (estimated VO2) test [22], treadmill duration test [22], exercise stress test [22], a modified sit-and-reach test [29], the Rockport Fitness Walking Test [29] and a submaximal cycle ergometry test [33]. In one study a sub-sample also wore an Actigraph to validate self-reported behavioral outcome findings [22].

Six studies included weight reduction as an outcome measure, reported as change in BMI, percentage body fat, body composition, waist circumference and waist-to-hip ratio. Four of the studies used objective ways of measuring weight changes, three by taking anthropometric measurements in a clinic (height, weight and/or body fatness and/or waist circumference) [19,23,33] and one using biomedical impedance analysis [29]. Two of the six studies used self-reported measures of weight and/or height [20,28].

All nine studies only using self-report measures of physical activity reported positive effects on physical activity outcomes, either between group effects [24,27,32,35] or within group effects over time [23,25,26,31,34]. Only half of the studies using more objective measures of physical activity found positive effects between groups [21,22] or within groups [19].

#### Retention rates

Studies reported retention rates for different timeframes making comparisons difficult. Retention rates were compared by considering post-test retention rates when reported, [19-22,24,27,28,32-35] and when not available the earliest post-intervention follow-up retention rate was used as the best approximation, the majority of which were short-term (< 3 months) [29-31], and a couple medium-term (3 ≤ months ≤ 6) [23,25].

Estimated retention rates ranged from 66–97%, ten of which were above 80% [20-22,25,28-30,32,33,35]. It must be noted the majority of these relatively higher retention rates were actual post-test measurements [20-22,25,28,32,33,35]. For the seven studies reporting retention rates lower than 80%, most were measured at post-test [19,24,27,34] with two based on approximations from reported post-intervention follow-up retention measurements: short-term [31], and medium-term [23], which may account for the relatively lower rates.

There were some commonalities between the studies with relatively high retention. All used highly motivated and/or self-select samples, eight of which had predominately female samples (range 66–100%) and five of the six studies reporting on education levels of participants, had predominately well-educated samples (range 71–95%). All but one study [30] were intended as multiple exposure interventions; the majority of which either used controlled program delivery, which provides new information at each exposure [21,22,25,28,29,33,35] and/or offered incentives to participants [20-22,25,28,30]. However these characteristics were not necessarily predictors of good retention. Due to the small number of heterogeneous studies reviewed we could not find any consistent relationship between retention rates and mode of delivery, intervention duration, intervention intensity and length of follow-up.

Three studies reported both retention rates at post-test and post-intervention follow-up retention rates, two of which were able to report rates above 80% at both times: one short-term post-intervention follow-up [29] and one at medium-term post-intervention follow-up [28]; and another study was able to report a post-test retention rate above 80%, for which the medium-term post-intervention follow-up retention rate was only slightly below at 77% [25]. All three interventions involved multiple exposures and used controlled program delivery.

### Study design: isolating the effect of the technology

Four studies isolated the effect of the computer-tailored intervention in terms of the technology by comparing to a tailored non-technology control group. Only one of these reported between-group differences in favor of the computer-tailored intervention on physical activity outcomes, however the intensity differed significantly between the groups [21].

### Intervention costs

Although most articles referred to the cost-effectiveness of computer-tailored interventions [19-21,23,25,27,29,31,32,35] only two reported on any basic economic measures such as costs. One Australian study provided an indication of the cost of a website delivered intervention, a face-to-face nutrition counseling intervention, and an Internet-based intervention with nutrition counseling [19]. They reported that although the original set-up costs for a website are costly (minimum $20 000 AUD) ongoing costs are minimal and additional cost savings may be had by participants due to no travel time or costs. One US study indicated operating costs of a computer-tailored telephone communications system was low, that is, between $1 to $2 US per call, including all personnel and non-personnel costs [35].

## Discussion

This narrative systematic review has described the range of evidence on 'second' and 'third' generation computer-tailored primary prevention interventions targeting physical activity behavior change in adults. Common characteristics of interventions that produced significant between-group effects and interventions with good retention rates were considered, as were internal and external validity of studies as measures of quality and generalizability.

This review differs from previous systematic reviews on computer-tailored interventions [5,6] in the following ways: our review was exclusive to primary prevention interventions; first generation computer-tailored interventions and studies in which tailoring was not generated through an expert system were excluded; the review was not limited to RCTs, but also included quasi-experimental studies and studies with limited interpersonal contact that did not involve counseling. To our knowledge, previous reviews have not attempted to gauge the external validity of such intervention studies, although they have included varying measures of external validity in their quality criteria. Doing so is important in determining their generalizability and relevance to health promotion practice [37].

The volume of evidence has grown since the publication of previous reviews which had indicated that the evidence of effectiveness of computer-tailored interventions in the promotion of physical activity was limited [5,6]. Several more recent studies reported significant positive effects either in comparison to a control group or over time, which boosts the overall evidence of efficacy. Overall, just over half of the studies reported positive short-to-medium term effects in comparison to a control group for physical activity behaviors or weight reduction, the majority of others reporting positive effects within the treatment group over time or positive effects on behavior mediators.

The efficacy of computer-tailored interventions is dependent on many factors such as the intervention quality, duration, exposure, intensity, use of theory, method of tailoring, source credibility and mode of delivery. Due to the small number of studies that isolated the effect of tailoring or the technology in their study design no conclusions can be drawn on their relative importance for success. Comparing participant's behavior to current recommendations, tailoring according to the participant's stage of change and tailoring feedback in more than one way were common in studies reporting significant positive between-group outcomes but using these tailoring methods was not necessarily predictive of success. More research is required to determine why and when tailoring is effective [5]. In agreement with the findings of a previous review [5] it seems that whilst the intervention should be based on theory, no one theory has proven to be more applicable or effective. Ensuring multiple intervention exposures may also be important but was neither necessary nor predictive of success.

The quality, intervention intensity, duration and mode of delivery differed widely for the seven computer-tailored interventions reporting significant positive between group effects on physical activity outcomes. Success of the intervention does not appear dependent on the technology used in its delivery or in its intensity, with little evidence that interventions of greater intensity had better outcomes. This was the case whether additional support was delivered through the technology or interpersonal communication. However very few studies compared intervention groups of differing intensity, a similar finding to that reported by a previous review [5]. Therefore there is insufficient evidence to determine the optimal intensity for computer-tailored interventions and to determine the best way of delivering interventions targeting more than one behavior change. More research is needed in this area [24,26].

It has been recommended that studies use a combination of validated self-reports with more objective measures of behavior change [5], however less than half the studies included objective measures of physical activity. It appears that the use of objective measures of physical activity may be important in determining whether self-reported changes found are actual, with only one third of studies using objective measures of physical activity finding positive between group effects on behavior.

The real-life effectiveness of such interventions is dependent on the external validity of studies, including the setting in which it was conducted, the characteristics and representativeness of the targeted and recruited population sample and methods of recruitment, all factors which influence the generalizability of findings to practice [37]. The external validity of reviewed studies was generally poor, resulting in uncertainty about such interventions' generalizability. This finding is not surprising given the majority of studies were RCTs as such designs aim to maximize internal validity and can sacrifice external validity, with results only generalizable to those participants who are willing to accept randomization [37]. A stronger focus on effectiveness and dissemination may assist in the development of programs in population-based effectiveness settings. Future RCTs should attempt to increase their external validity by including representative participants and answering real-world questions [37]. However this review found such characteristics of design lacking, with the common use of either small, homogenous or unrepresentative samples, restrictive exclusion criteria and for some a lack of comparison conditions relevant to real-world decisions, that is, comparison to no treatment controls only. Such characteristics significantly limit the dissemination of such interventions into practice [18].

Although determining cost-effectiveness was not the purpose of this review we recommend future studies at the very least report on basic economic measures such as costs, which are relevant to decision-makers and can assist in intervention uptake, dissemination and inform more advanced cost-effectiveness studies [18,37]. Cost-effectiveness analyses are recommended as they will be important in determining the additional value of such intervention delivery modes over the more traditional delivery modes such as face-to-face counseling. The presumed cost savings for participants due to no travel time or costs may be particularly important for those living in rural or isolated areas.

There was a fundamental lack of long-term post-intervention follow-up, with only one study demonstrating intervention effects were maintained at two years post-baseline[26]. However the generalizability of this study's findings and application to practice may be limited. More studies with long term follow-up of 12 months post-intervention are needed [25].

Previously noted poor retention rates of computer-tailored interventions, in particular web-based interventions [5-7,11] prompted consideration of characteristics of interventions that might maintain engagement and retention such as the intervention's interactivity, duration, intensity, setting and study sample characteristics. However with the small number of studies comparing retention rates became problematic due to their varied follow-up length and therefore we could not form any definite conclusions. However based on our findings and other published reviews it seems the following intervention characteristics may be important in enhancing participant retention: ensuring multiple exposures to the intervention material, preferably evolving intervention materials or using controlled program delivery; the use of incentives; prompts through another medium; interactive and dynamic web components; and individualized tailoring [6,11,31]. Each of these characteristics may be insufficient on its own to result in good retention and therefore all will need to be considered in intervention design, sample size calculations and probable retention rates in the future.

Engaging and retaining interest using the Internet and email mediums, which are increasingly busy and through which many other information sources compete for attention will be a challenge. Attracting people to return to a website is challenging in particular for websites that do not offer new information at each visit [7]. Telephone support in Internet-based interventions as an intervention strategy and a maintenance strategy has been shown to be as effective as face-to-face contact and to result in greater adherence and maintenance to the intervention and thus future research in this area is warranted as a way of increasing Internet engagement [25].

The limitations of this review must also be acknowledged. Firstly, this review did not actively seek unpublished studies, although one such study was included. Therefore when considering the findings of this review, the possibility of publication bias must be noted, resulting in a bias of studies with positive findings. However given the fairly high proportion of published studies reviewed that did not have significant findings, it is believed that the likelihood of publication bias is minimal.

Secondly, this review did not include articles in which physical activity behavior was not a primary outcome. This meant that articles were excluded in which psychological indicators, behavior mediators or process measures were the only outcome measures reported. Although behavior mediator effects, where available, were examined when behavioral change outcome effects were absent or conflicting, process measures were not described. This limits our discussion on retention, engagement and acceptability of computer-tailored interventions and their components in different population subgroups and settings. Although this was not the purpose of the review, reviewing research in this area would be worthwhile as it may indicate different levels of acceptance and the relative effectiveness in different population subgroups. This may be particularly important given the majority of reviewed studies had predominantly female, Caucasian, well educated samples.

Thirdly, we have not attempted to estimate a pooled effect size or to calculate and compare effect sizes of different studies due to the heterogeneity of studies in terms of their intervention design, delivery method, exposure and intensity, participants, study design and methods, and outcome measures. Such factors make such comparisons difficult [38] and inadequate [13]; hence a narrative systematic review was conducted. The two previous reviews on computer-tailored health behavior interventions most relevant to this review reported small to medium effect sizes [5,6]. We agree with these and other authors that despite the small effect sizes found, such interventions can have substantial impact at a population health level, with their potential for wide distribution at low cost [5,6]. However it will be critical to determine whether such findings are generalizable, can be replicated and to ensure adequate reach and engagement within varied population groups for such interventions.

In addition, our findings on common characteristics of successful interventions and those with good retention are limited due to the small number of heterogeneous studies included and our reliance on varying levels of detail provided in each article. Only a small proportion of the retrieved articles were included in this review. The main reasons for this include: many studies were duplicated in the databases that were searched, broad search terms were used and the exclusion criteria were specific and detailed. For example, the search terms did not distinguish between first, second and third generation interventions and first generation interventions which make up a substantial proportion of the literature were not considered in this review.

Lastly, due to the small number of studies reporting positive weight reduction outcomes, the relative contribution of nutrition and physical activity behaviors to such outcomes were not examined in this review.

Future research should endeavor to replicate studies in different populations to indicate effectiveness and generalizability. Following the example of Vandelanotte and colleagues where the same theory-based intervention was trialed and adapted in different population groups & settings and followed up long term [23,24,26,27,39] in addition to their reports on the acceptability and feasibility of these interventions in individuals of different age, sex, education level and computer literacy [9,10] is important in building the evidence base.

## Conclusion

The evidence base regarding the effectiveness of computer-tailored physical activity interventions is growing. However, no conclusions on their effectiveness can be drawn, given inconsistent results of the studies. These interventions have the potential to reach large groups of people, albeit self-selected groups. The uncertainty lies in whether the reported behavior changes found can be sustained long-term, and whether they are generalizable. Also, the relative success of different components of efficacious interventions is unclear in addition to the optimal intervention intensity. Interventions should be based on theory and ensure multiple exposures, preferably with evolving intervention materials. These factors may also be important in enhancing retention, in addition to tailoring, the use of incentives, interactive and dynamic components and prompts through another medium.

Further research will be needed on computer-tailored physical activity primary prevention interventions including: the replication of successful efficacy trials in different settings and population groups; more effectiveness studies in representative heterogeneous populations that compare to current practice; a review of the research on engagement and acceptability of such interventions; and most importantly long-term post-intervention follow-up and cost-effectiveness studies. More research is also needed to determine the optimal intensity for population-level interventions.

## Competing interests

The authors declare that they have no competing interests.

## Authors' contributions

LN participated in the design of the study, reviewed the literature and drafted the manuscript. BOH participated in the design of the study, reviewed the literature and revised and edited the manuscript. AM conceived of the study, participated in its design and coordination, and revised and edited the manuscript.

## Supplementary Material

Additional file 1 **Table S1: Summary of physical activity behavior change interventions**. The data provides a summary of each study regarding the following areas: Context/setting and sample characteristics; Intervention characteristics & control condition; Study design & evaluation Method; Outcome measures; and Key Findings. Click here for file
