# Supplementary material for: Computer-tailored physical activity behavior change interventions targeting adults: a systematic review
Source: Int J Behav Nutr Phys Act. 2009 Jun 3;6:30. doi: 10.1186/1479-5868-6-30 (PMC2700068; doi:10.1186/1479-5868-6-30)
Supplement: Additional file 1 — Table S1: Summary of physical activity behavior change interventions. The data provides a summary of each study regarding the following areas: Context/setting and sample characteristics; Intervention characteristics & control condition; Study design & evaluation Method; Outcome measures; and Key Findings. [file 1479-5868-6-30-S1.doc]

## Table S1: Summary of physical activity behavior change interventions

| Study & Focus | **Context/setting and sample characteristics** | **Intervention characteristics & control condition** | **Study design & evaluation Method** | **Outcome measures** | **Key Findings** |
| --- | --- | --- | --- | --- | --- |
| **Booth (2008) [19]**  Australia  **Other behaviors targeted:** NU & weight | **Setting:** community  **Recruitment:** local and city newspapers, flyers in local community centers, libraries & health centers  **Eligibility/inclusion criteria:** 24.5 ≤ BMI ≤ 37; Internet access  **Exclusion criteria:** <18y, pregnant/lactating, receiving medications for Type 1/2 DM  **Participants (baseline):** 73 adults  **Retention rate:** 73%  **Final sample characteristics:** 79%F; 81% Anglo-Australian; 51% tertiary education | **G1:** CT Internet exercise program  **G2:** CT Internet diet + exercise program (as per G1 plus diet program & minimum 3 diet emails)  **Tailoring:** current recommendations, previously set goals  **Theory:** Goal-setting theory  **Frequency:** multiple exposure (weekly at minimum)  **Duration:** 12 weeks  **Incentives offered:** No | **Design:** Pilot randomized trial; randomized by individual; groups comparable at baseline (demographics & OM)  **Follow-up:** 12 weeks (PT) | **Primary OM:** anthropometric measurements (weight, height, waist circumference), frequency & intensity of PA in previous week, average daily no. steps  **Instrument:** PA questionnaire (based on Active Australia survey), pedometer.  **Validated:** Yes | **Behavior:** Significant increase in no. daily steps taken from baseline to follow-up, but no difference between groups; No significant differences in other self-reported PA  **WR:** Significant fall in waist circumference & BMI in both groups, no differences between groups; 21% participants moved from having waist circumference in very high risk category to a lower risk category; Significant association between step change & waist circumference change from week 1 to 12 for both groups |
| **Cook (2007) [20]**  United States  **Other behaviors targeted:** NU | **Setting**: workplace  **Recruitment**: email letter, online flyer, posters  **Eligibility/Inclusion criteria:** workplace employees (n=~5000) in 3 offices of a human resources co.  **Exclusion criteria:** NR  **Participation rate**: 10%  **Participants (baseline):** 480 adults (G1= 247; G2 = 233)  **Retention rate:** 87% (G1=85%; G2 = 87%)  **Final sample characteristics:** 72%F, 81% White; 95% college or higher education; mean age: G1=41.99y; G2 = 42.03y | **G1:** CT Internet ‘Health Connection’ program (graphics, audio & video)  **G2:** Generic print materials on same topics (5 commercially available booklets)  **Tailoring:** stage of change, current recommendations  **Theory:** SCT & TTM, health behavior change theory  **Frequency:** multiple exposure  **Duration:** 3 months  **Incentives offered**: Yes | **Design:** RCT; randomized by individual; online questionnaire; baseline groups equivalent (demographics & OM)  **Follow-up:** 3 months (PT) | **OM:** frequency of PA in past week; motivation to improve exercise; behavioral intentions towards exercise; exercise self-efficacy; stage of change for weight and exercise; weight; process evaluation measures  **Instrument:** online health survey consisting of many measurement items  **Validated:** Majority have been pre-tested & validated, validation of some UK. | **Behavior:** No difference between two groups in increasing PA; both groups improved significantly from pre- to post-test for PA OM;  **WR:** No significant differential change in weight between two groups. |
| **Hurling (2007) [21]**  United Kingdom | **Setting:** Community  **Recruitment:** NR, occurred through market research recruitment agency  **Eligibility/Inclusion criteria:** living within 50km study centre; 30-55 yrs; BMI 19-30; not vigorously active; not taking regular prescription medication; Internet & email access; mobile phone user; not employed by Unilever.  **Exclusion criteria**: heart condition, pain in chest when exercising, joint problem that might be aggravated by exercise  **Participants (baseline):** 77 adults(G1= 47; G2= 30)  **Retention rate:** 96%  **Baseline sample characteristics:** 66%F; mean age 40.4y (SD=7.6); mean BMI 26.3 (SD=3.4) | **G1:** CT intervention delivered through Internet, email and mobile phone  **G2:** verbal advice on recommended PA levels on one centre visit  **Tailoring:** peer behavior, previously set goals, current recommendations, perceived barriers  **Theory:** social psychological theories (social comparison, decisional balance, elaboration likelihood, Goal)  **Frequency:** multiple exposure (weekly at minimum)  **Duration:** 9 weeks  **Incentives offered:** Yes | **Design:** RCT; randomized by individual (after being stratified by age, gender, BMI); groups comparable at baseline (demographics & OM); anthropometric measures at clinic; Participants instructed in use of system & issued a wrist-worn accelerometer & blue tooth compatible mobile phone in one centre visit; Baseline data = 3 weeks worth PA data; Intention to treat analysis conducted.  **Follow-up:** 10 weeks (PT) | **Primary OM:** change in moderate PA; weight change, % body fat, resting blood pressure;  **Instrument:** longer version of IPAQ & Bluetooth connected wrist-worn accelerometer  **Validated:** Yes  **Other OM:** Motivation, skills used to increase PA  **Instrument:** Exercise ‘Skills and Knowledge Questionnaire’ | **Behavior:** Higher level of MI-PA in G1 than G2: G1 increased on average 2h18min MI-PA/week over G2; Reduction in weekly hrs spent sitting: significant difference between G1 & G2;  **WR:** Significant difference between G1 & G2 for change in % body fat  **Mediators:** G1 reported a significantly greater increase over baseline than G2 for perceived control and intention to exercise; G1 rated themselves as more satisfied with their fitness & well-being; and had significantly higher sense of internal control and external control over exercise than G2 |
| **Marcus (2007) [22]**  United States | **Setting:** community  **Recruitment:** newspaper advertisements, local hospital worksite’s Intranet, email and employee pay stubs  **Eligibility/inclusion criteria:** healthy, age 18-65y, under active (defined as participating in ≤ 90 min/wk moderate or vigorous PA);  **Exclusion criteria:** BMI>35, certain medical, psychiatric conditions & medication, >3 alcoholic drinks/day on ≥5 days/wk  **Participants (baseline):** 239 sedentary adults (G1 = 80, G2 = 81, G3 = 78)  **Retention rate:** 6 months 91% (G1 = 94%, G2 = 88%, G3 = 92%); 12 months 86% (G1 = 88%, G2 = 82%, G3 = 89%)  **Sample characteristics (baseline):** 82%F; 90% Caucasian; mean age 44.5y, 71% college education or above | **G1:** CT intervention delivered via telephone  **G2:** CT intervention delivered through print  **G3:** wait-list control  **Tailoring:** stage of change  **Theory:** TTM, SCT  **Frequency:** 14 contacts (more frequent at beginning of study i.e. weekly in 1^st^ month) each call approximately 13 min  **Duration:** 12 months  **Incentives offered:** Yes | **Design:** RCT; randomized by individual; groups comparable at baseline (demographics & OM); Participants completed physical activity log & questionnaire monthly; Interviewer-administered; Intention to treat approach taken for analysis  **Follow-up:** 6 & 12 months (PT) | **Primary OM:** PA participation  **Instrument:** 7-day Physical Activity Recall interview  **Validated:** Yes  **Other OM:** anthropometric measurements, Functional capacity (estimated VO_2_) at maximal heart rate, treadmill duration, stages of change  **Other instruments:** exercise stress test, psychosocial questionnaires; A sub-sample (30%) also wore an Actigraph to validate self-reported PA outcome findings  **Validated:** Yes | Overall individually tailored print & telephone interventions efficacious in PA adoption, print more so for PA maintenance  **6 months:**  **Behavior:** G1 & G2 significantly greater increase than G3 participants in PA mins/wk but no difference between G2 & G3; G1 & G2 significantly more likely than G3 to achieve 150min PA/wk  **Mediators:** Significant between-group differences in behavioral processes, cognitive processes, decisional balance & self-efficacy; G1 & G2 more likely to report increase than G3  **12 months:**  **Behavior:** G2 significantly greater increases in PA min/wk than G3, G2 significantly better than G1 (G2 increased, G1 decreased); G2 participants significantly more likely to achieve 150min PA/wk than G3  Exercise stress test: no significant differences at 6 or 12 months  **Mediators:** as per 6 months; self-efficacy increased for G2 participants and declined for G1 participants |
| **Spittaels (2007) [23]**  Belgium | **Setting:** workplace  **Recruitment:** email, posters, internal newsletters.  **Response rate:** 7%  **Eligibility/Inclusion criteria:** Age 25-55y, Internet access.  **Exclusion criteria:** history CVD  **Participants (baseline):** 526 adults  **Retention rate:** 72% (G1= 66%; G2= 69%; G3=1 79%)  **Baseline sample characteristics:** 69.4%M; mean age 39.5y; 61.7% college or uni degree | **G1:** CT intervention via Internet & stage-based reinforcement e-mails  **G2:** CT intervention via Internet  **G3:** non-tailored Internet intervention  **Tailoring:** current recommendations, stage of change, self-efficacy, attitudes, intentions  **Theory:** TTM & Theory of Planned Behavior  **Frequency:** multiple exposure (at minimum 5 emails)  **Duration:** 6 months (emails for 8 week period)  **Incentives offered:** Yes | **Design:** RCT; baseline groups comparable (demographics & OM); paper questionnaires  **Follow-up:** 6 months (post-baseline) | **Primary OM:** frequency & duration of PA (at work, as transportation, for household chores, during leisure time) & daily sitting time); additional objective WR assessment in sub sample (1 worksite, n=57): height, weight, body fatness, blood pressure, heart rate and PA accelerometer  **Instrument:** Long usual week version of the IPAQ  **Validated:** Yes  **Other OM:** process evaluation measures; additional objective assessment (height, weight, body fatness, blood pressure, heart rate and PA accelerometer) in 1 of the 6 worksites (n = 57) | **Behavior (total sample):** no significant difference between groups however all 3 groups reported significant increase in PA;  **WR (for sub sample, n=57):** significant time x group interaction for % body fat – greater decline in % body fat in G1 compared with other 2 groups  -significant decrease in BMI, fat %, diastolic blood |
| **Spittaels (2007) [24]**  Belgium | **Setting:** Community  **Recruitment:** distributed brochures (school)  **Eligibility/Inclusion criteria:** 20-55 yrs, Internet access.  **Exclusion criteria:** history of CVD  **Participants (baseline):** 434 parents and staff of 14 primary & secondary schools (G1 = 173, G2 = 129, G3 = 132)  **Retention rate:** 66%  **Baseline sample characteristics:** 66%F; mean age 41.4y (SD = 5.6); mean BMI 24.6 (SD = 3.6), 66.8% higher education, 86.1% employed  **Final sample characteristics:**  Men, participants with higher BMI & dropouts significantly higher in intervention groups (40%) than control group (21%) | **G1:** CT & non-tailored Internet intervention; 7 non-tailored emails, repeated tailored feedback possibility at 3 months  **G2:** CT & non-tailored Internet intervention (single exposure)  **G3:** wait list control  **Tailoring:** current recommendations, stage of change, self-efficacy, attitudes, intentions  **Theory:** TTM & the Theory of Planned Behavior  **Frequency:** multiple exposure (minimum 7)  **Duration:** 6 months  **Incentives offered:** Yes | **Design:** RCT; potential participants randomized by school region; Both intent-to-treat and retained sample analysis conducted.  **Follow-up:** 6 months (PT). | **Primary OM:** frequency & duration of PA (at work, as transportation, for household chores, during leisure time) & daily sitting time;  **Instrument:** Long usual week version of the IPAQ  **Validated:** Yes  **Other OM:** Process evaluation measures | **Behavior:** Participants in both groups 1 & 2 reported a significant increase in PA level and decrease in time spent sitting compared to G3; Significant time by group effects were found for active transportation, PA in leisure time and time spent sitting on a weekday.  Intention to treat analysis: intervention resulted in a significant increase of 10% of participants that met PA recommendations in G1 compared to non-significant increases of 5% (G2) & 4% (G3). |
| **Steele (2007) [25]**  Australia | **Setting:** community  **Recruitment:** local newspaper advertisements  **Eligibility/Inclusion criteria:** ≥18y; functionally mobile ≥10 mins; inactive; Internet access; signed informed consent.  **Exclusion criteria:** No medical clearance from participant’s general practitioner if identified as having cardiac & other health problems at screening  **Participants (baseline):** 192 inactive adults (G1 =65, G2 = 65, G3 = 62).  **Retention rate:** PT 83%; PI follow-up 77% (G1=80%; G2=72%; G3=77%)  **Sample characteristics (baseline):** 83%F; mean age 38.7+/-12y; mean BMI 32.1 +/-7.5 | **G1:** Face-to-face intervention with trained program facilitator  **G2:** CT Internet intervention, weekly emails & opportunity to attend 2 (1hr) face-to-face sessions  **G3:** CT Internet intervention & weekly emails  G2 & G3 had access to on-line Health-eSteps rep, Nutritionist & Exercise Physiologist via email  **Tailoring:** unclear, noted individualized & personally relevant  **Theory:** SCT & self-management framework.  **Frequency:** multiple exposures (minimum weekly)  **Duration:** 12 weeks  **Incentives offered:** Yes | **Design:** RCT; randomized by individual; groups comparable at baseline (demographics & primary OM); face-to-face contact for data collection; each participant provided with pedometer; intention-to-treat analysis conducted; testing of statistical equivalency conducted  **Follow-up:** 5 months PI | **Primary OM:** frequency & duration of PA  **Instrument:** Active Australia questionnaire  **Validated:** Yes  **Other OM:** height, weight PA self-efficacy, Internet self efficacy; social support for PA, process measures, pedometer usage | **Behavior:** Mean minutes PA increased by 270 min (G1), 177 min (G2) & 170 min (G3); no group by time interaction; All groups significantly increased PA from baseline to 5-month follow-up but G3 decreased between 2 & 5 months. |
| **Vandelanotte (2007 & 2005) [26,27]**  Belgium  **Other behaviors targeted:** NU | **Setting:** University computer laboratory, controlled  **Recruitment:** local media, posters, leaflets and email. **Eligibility/Inclusion criteria:** Age 20-60y  **Exclusion criteria:** medical complaints related to PA or fat intake.  **Participants (baseline):** 1023 adults  **Retention rate:** 6 months 75%; PI follow-up38%  **Final sample characteristics (6m):** 65%F; mean age 39.1Y+/-9.6; 70% higher education; 86% employed; mean BMI 24.5 +/-4.1; Men & younger participants more likely to drop out | G1-G3 received interactive CT intervention delivered through desktop computer application  **G1:** tailored PA and fat intake interventions simultaneously  **G2:** tailored PA intervention at baseline and tailored fat intake intervention 3 months later  **G3:** tailored fat intake intervention at baseline and tailored PA intervention 3 months later  **G4:** wait-list control; received tailored interventions at 6 months.  **Tailoring:** current recommendations, stage of change, self-efficacy, attitudes, intentions  **Theory:** Theory of Planned Behavior & TTM  **Frequency:** single-exposure  **Duration:** 6 months  **Incentives offered:** Yes | **Design:** RCT; randomized by individual; mail questionnaires.  **Follow-up:** 6 months (PT), 2 years post-baseline (follow-up study in which control group omitted from analysis as were wait list) | **6-months:**  **OM:** frequency & duration of PA (at work, as transportation, for household chores, during leisure time) & daily sitting time; **Instrument:** Long self-administered version of the IPAQ  **Validated:** Yes  **2 year follow-up:**  **OM:** total PA; mod+high intensity PA (Groups 1 & 2 only included for PA analysis, n=257); | **6 months:** Groups 1-3 had significantly higher PA scores (total PA) compared to G4; There was no significant difference between the sequential and simultaneous intervention groups for PA  **2 years (no control group):**  No differences in PA change between Groups 1 & 2 but strong time effects for both total PA & mod+high intensity PA for total group |
| **Winett (2007) [28]**  United States  **Other behaviors targeted:** NU | **Setting:** churches  **Recruitment:** churches through letter & phone; individual church members through announcements, flyers, posters, bulletins & luncheons  **Participation rate:** 14 of 23 churches  **Eligibility/inclusion criteria:** members of consenting churches  **Exclusion criteria:** certain medical conditions necessitated medical clearance before participating in PA component  **Participants (baseline):** 1071 church members [(G1 = 364 (5 churches), G2 = 364 (5 churches), G3 = 343 (4 churches)]  **Retention rate:** PT 89% (G1=91%; G2 = 85%; G3 = 87%); PI follow-up 87% (G1= 90%; G2 = 85%; G3 = 85%)  **Baseline sample characteristics:** 33%M, median age 53y; 23% African American, 57% BMI≥25, 60% sedentary (<7500 steps/day) | **G1:** CT Internet intervention (through church) & additional church-based support  **G2:** CT Internet intervention (through church)  **G3:** waitlist control  **Tailoring:** current recommendations, previously set goals  **Theory:** SCT  **Frequency:** multiple exposure, weekly modules successively available  **Duration:** 12 weeks  **Incentives offered:** Yes | **Design:** Group Randomized Trial; randomized by church (after being stratified by denomination, size and primary racial background of members);  pragmatic analyses conducted; unequal % of African-American participants across groups  **Follow-up:** 12 weeks (PT) & 6 months PI | **Primary OM:** steps, weight, height  **Instrument:** Physical Activity Readiness Questionnaire, Veterans Specific Activity Questionnaire, pedometer,  **Validated:** Yes  **Other OM:** social support, self efficacy, outcome expectations, self-regulation variables, process measures  **Instrument:** The Health Beliefs Survey, log-ins | **12 weeks:**  **PA:** Significantly greater increase in steps between G1 & G3  **WR:** G1 participants lost small amount weight & compared to small weight gain in G3 participants difference was significant; marginally significant difference between G2 & G3; no difference between G1 & G2.  **Mediators:** G1 &G2 made greater changes in PA self-regulation behaviors compared to G3  **6-months:**  **PA:** G1 participants significantly more likely to reach step goals than G3 participants  **WR:** no differences between groups 1, 2 & 3  **Mediators:** similar effect observed as per PT |
| **Hageman (2005) [29]**  United States | **Setting:** community  **Recruitment:** newspaper advertisement  **Eligibility/Inclusion criteria:** women aged 50-69y, English speaking, access to home computer with Internet capacity, answered No to all Q on PA Readiness Questionnaire  **Exclusion criteria:** participating in 30 min MI-PA ≥5 days/week over a 6 month period  **Participants (baseline):** 31 women (G1 = 15, G2 = 16)  **Retention rate:** PT 97%; PI follow-up 90%  **Sample characteristics (baseline):** 100%F; majority White (G1 86.7%, G2 = 100%); some college, graduate or higher (G1 86.6%, G2 = 81.4%); employed (G1 80%, G2 = 50.1%) | **G1:** CT Internet newsletters consisting of 5-7 brief articles  **G2:** 3 generic Internet newsletters  **Tailoring:** previous behavior, previously set goals, perceived benefits & barriers, self-efficacy  **Theory:** Pender’s Health Promotion Model (based on SCT)  **Frequency:** multiple exposure (3 occasions, monthly), low intensity  **Duration:**2 months  **Incentives offered:** No | **Design:** RCT; randomized by individual; UK whether groups significantly different at baseline (demographics or OM)  **Follow-up:** 3 months post-baseline (1 month PI) | **Primary OM:** time engaged in PA & daily energy expenditure, cardiorespiratory fitness, body composition, flexibility  **Instruments:** Modified 7-Day Activity Recall, Rockport Fitness Walking Test, biomedical impedance analysis, modified sit-and-reach test  **Validated:** Yes  **Other OM:** perceived benefits & barriers to PA; self-efficacy for PA, process measures  **Other Instruments:** 29-item Benefits Scale, 14-item Barriers Scale of the Exercise Benefits/Barriers Scales; Self-efficacy for Exercise Habits Scale  **Validated:** Yes | **Behavior:** Neither group increased in self-reported PA time. Both groups improved in flexibility  **WR:** Significant time by group interaction for VO_2_ max & % body fat: VO_2_ max & % body fat decreased in G2 & did not change in G1 (conflicting results)  **Mediators:** both groups declined in perceptions of barriers to PA; Significant time by group interaction for self-efficacy: increased in G2 and decreased in G1 |
| **Kypri (2005) [30]**  New Zealand  **Other behaviors targeted:** NU, alcohol consumption & smoking | **Setting:** primary care  **Recruitment:** invited in person by research assistant  **Eligibility/inclusion criteria:** attending primary care at university  **Exclusion criteria:** NR  **Participants (baseline):** 218 young adults attending student health service of university  **Retention rate:** 86%  **Baseline sample characteristics:** 49%F; mean age 20.2y (SD = 1.5); 75% European, 8% Maori | **G1:** CT intervention via desktop computer program  **G2:** computerized assessment only  **G3:** no treatment control  **Tailoring:** current recommendations, peer behavior  **Theory:** NR  **Frequency:** single exposure (1 computer session)  **Duration:** 6 weeks  **Incentives offered:** Yes | **Design:** RCT; participants assigned computerized random number generator in blocks of 15 (5 per group); baseline questionnaire completed in clinic; follow-up - web-based survey  **Follow-up:** 6 weeks PI | **Primary OM:** PA in past week (no. episodes, duration, level of exertion)  **Instrument:** computerized questionnaire  **Validated:** No | **6 weeks:** G1 had significantly greater compliance with PA recommendations than G3 |
| **Marshall (2003) [31]**  Australia | **Setting:** workplace  **Recruitment:** NR  **Eligibility/inclusion criteria:** academic (faculty) & general staff at an Australian regional university; access to email  **Exclusion criteria:** NR  **Participation rate:** 46%  **Participants (baseline):** 655 university staff (G1 = 327, G2 = 328)  **Retention rate:** 78% (G1 = 76%, G2 = 80%)  **Sample characteristics:** 49%M; mean age 43y (SD=10); 70% ≥post/secondary education; mean BMI 24 (SD=7) | **G1:** CT Internet intervention (similar content to booklets received by G2) & 4 stage-based emails  **G2:** stage-targeted letter & booklet, 4 behavioral reinforcement letters (similar content to G1 emails)  **Tailoring:** stage of change  **Theory:** TTM  **Frequency:** multiple exposure (every 2 weeks at minimum)  **Duration:** 8 weeks  **Incentives offered:** No | **Design:** RCT; randomized by individual (after stratification by stage of change); groups comparable at baseline (demographics); Data collected using CATI; intention to treat analysis conducted  **Follow-up:** 10 weeks post-baseline | **Primary OM:** change in self-reported PA (frequency & duration of vigorous intensity, moderate intensity & walking activity, time spent sitting over past 7 days, cycling to/from work, walking during work breaks)  **Instrument:** IPAQ Short Past 7 day  **Validated:** Yes  **Other OM:** stage of change, process measures | **Behavior & mediators:** no significant differences between or within groups for PA or stage of change  **Sub-group analyses:** For those participants inactive at baseline the following significant within-group differences were found: G1 participants had decrease in time reported sitting on a weekday; G2 participants had significant increase in total PA |
| **Napolitano (2003) [32]**  United States | **Setting:** Workplace  **Recruitment:** print, in-person & electronic channels  **Eligibility/inclusion criteria:** hospital employees (US), aged 18-65 yrs, engaging in 120 mins or fewer moderate intensity PA/wk or 60 mins or fewer vigorous intensity PA/wk.  **Exclusion criteria:** certain medical or psychiatric conditions, alcoholism or other substance abuse, current/planned pregnancy, being too active  **Participants (baseline):** 65 hospital employees (G1= 30; G2= 35)  **Retention rate:** 80% (G1= 70%; G2= 89%)  **Sample characteristics (baseline):** 86%F; mean age 42.8y (SD=10); 91% White; 78% highly educated;  **Sample characteristics (follow-up):** More men than expected dropped out of study. More participants in intervention group dropped out than in control group. | **G1:** CT & non-tailored Internet intervention & email tip sheets  **G2:** wait list control  **Tailoring:** stage of change  **Theory:** SCT, TTM  **Frequency/intensity:** multiple exposure to static website (weekly at minimum by email)  **Duration:** 12 weeks  **Incentives offered:** No | **Design:** RCT; randomized by individual; telephone assessments; groups comparable at baseline (demographics & OM);  **Follow-up:** 1& 3 months (PT) | **Primary OM:** PA  **Instruments**: Physical Activity Readiness Questionnaire (PAR-Q), Physical Activity Stage of Change and the Behavioral Risk Factor Surveillance System (BRFSS)  **Validated:** Yes  **Other OM:** questions regarding computer usage, comfort & skill with using Internet & email.  **Instruments:** NR | **1 month (n=57):**  **Behavior:** G1 exhibited higher levels of moderate mins of PA and of walking mins than G2.  **Mediators:** G1 significantly more likely to have progressed in stage of motivational readiness than in G2;  **3 months:**  **Behavior:** Differences between groups in walking minutes still significant  **Mediators:** Participants in G1 significantly more likely than those in G2 to progress in stage of motivational readiness when compared to baseline |
| **Veverka (2003) [33]**  United States  **Other behaviors targeted:** NU, weight reduction | **Setting:** military  **Recruitment:** advertisements, referrals & personal contact  **Eligibility/Inclusion criteria:** Age 30-44y, not enrolled in any mandatory fitness/weight management program, ≥1y active military service  **Exclusion criteria:** health conditions requiring medication that could alter plasma lipid levels/cardiac function  **Participants (baseline):** 42 air force enlisted men  **Retention rate:** PT 93% (G1 = 20, G2 = 19) (with reduced sample for some OM)  **Baseline sample characteristics:** 100%M; ~1/3 participants in each group well-trained with above average fitness levels | **G1:** CT intervention via Internet newsletter  **G2:** no treatment control  **Tailoring:** stage of change  **Theory:** TTM  **Frequency:** multiple exposure (encouraged at least once/month); low intensity  **Duration:** 6 months  **Incentives offered:** No | **Design:** RCT; randomized by individual; physiological measures taken at Air force base clinic laboratory; groups comparable at baseline (OM); reduced sample for cholesterol & bike score)  **Follow-up:** 6 months (PT) | **Primary OM:** cardiorespiratory fitness (VO_2_), BMI (height, weight), waist-to-hip ratio, body fat %, cholesterol, blood pressure, heart rate  **Instrument:** submaximal cycle ergometry testing, other anthropometric measurements  **Validated:** Yes | **Behavior:** no difference in VO_2_ for G1 over time;  **WR:** G1 participants significantly more likely to improve in weight, BMI, % body fat, waist-to-hip ratio & resting heart rate than G2 participants; for diastolic blood pressure & total blood cholesterol there was a significant treatment by pre-test score interaction i.e. those needing treatment at pre-test improved in these measures whereas those with normal levels at pre-test did not improve. |
| **Hager (2002) [34]**  United States | **Setting:** workplace  **Recruitment:** interoffice mail  **Eligibility/inclusion criteria:** university staff  **Exclusion criteria:** NR  **Participants (baseline):** 525 university staff (G1 = 175, G2 =175, G3 = 175)  **Retention rate:** 77% (G1 = 76%, G2 = 77%, G3 = 177%)  **Baseline sample characteristics:** 56%F, 94% Caucasian with some college education, mean age 42y (SD=6.39) | **G1:** CT Internet intervention (stage-based) & 5 stage-based emails  **G2:** CT Internet intervention (action-message) & 5 action & maintenance stage emails  **G3:** 5 nutrition email messages  **Tailoring:** stage of change  **Theory:** TTM  **Frequency:** single exposure to website, weekly emails  **Duration:**6 weeks  **Incentives offered:** No | **Design:** RCT; randomized by individual; Participants completed an online questionnaire at baseline & follow-up; groups comparable at baseline (demographics) with exception of gender; conducted separate gender analyses  **Follow-up:** 6 weeks (PT) | **Primary OM:** daily PA, leisure-time activity, occupational activity  **Instrument:** online version of Seven-day PA Recall questionnaire, Health Insurance Plan of New York Questionnaire  **Validated:** Yes  **Other OM:** self-efficacy | **Behavior:** G1 & G2 had significant increases in leisure activity from pre- to post-test. G2 showed significant increases in kilocalories/day of energy expenditure (7 day PA) and occupational activity; no between group differences apart from gender analysis (see below);  **Mediators:** Each group significantly increased in level of stage of change from pre- to post-test, no difference between groups  **Gender analyses:**  G2 males significantly greater increase in kilocalories/day of energy expenditure compared to G1.  G1& 2 females had significant increases in leisure activity from pre- to post-test |
| **Pinto (2002) [35]**  United States  **Other behaviors targeted:** NU [36] | **Setting:** community  **Recruitment:**  letter through medical practice  **Eligibility/Inclusion criteria:** patients of a multi-site, multi-specialty group practice in Eastern Massachusetts  **Exclusion criteria:** <25 years, existing medical or psychological condition, engaged in regular MI-PA/VI-PA, did not have ‘suboptimal’ diet  **Participants (baseline):** 298 sedentary adults with ‘suboptimal’ diet (G1= 148; G2= 150).  **Retention rate:** 3 months 84%; 6 months 81.5%  **Baseline sample characteristics:** 72%F; mean age 45.9y; 85% employed; 45% White; 45% African-American; 71.4% ≥13y education | **G1:** CT NU intervention via automated telephone counseling system & printed status reports  **G2:** CT PA intervention via automated telephone counseling system & printed status reports  **Tailoring:** stage of change, current recommendations  **Theory used:** SCT, TTM & Decision-making theory  **Frequency:** multiple exposure (weekly for first 3 months and at least bi-weekly thereafter)  **Duration:** 6 months  **Incentives offered:** No | **Design:** RCT; randomized by individual; baseline groups equivalent (demographics & PA OM); Analyses controlled for age, gender, race & baseline intake.  Intention to treat analyses; Assessments conducted at home visit for baseline then by telephone (CATI) at subsequent measurement times  **Follow-up:** 3, 6 months (PT) | **Primary OM:** Self-report PA behavior (occupational and leisure PA, hours spent in sleep, MI-PA, VI-PA, caloric expenditure); meeting recommended PA levels, Motivational readiness for PA  **Instrument**: 7-day Physical Activity Recall; Stage of Motivational Readiness for PA  **Validated**: Yes  **Other OM**: Process measures | **3 months:**  **Behavior:**  G2 significantly more likely to meet recommendations for MI-PA or VI-PA than G1; significant effect of intervention on daily kilocalorie expenditure in MI-PA for G2;  **Mediators:**  In G2 there was a significantly greater no. intervention subjects in Action stage, significantly fewer participants in contemplation stage than G1;  **6 months:**  **Behavior:** None of the above findings were maintained |

BMI = Body Mass Index; CATI = Computer Assisted Telephone Interviewing; CT=Computer-tailored; G = Group; PA = physical activity; IPAQ = International Physical Activity Questionnaire; MI-PA = moderate intensity physical activity; NU = nutrition; OM = Outcome Measure; PI=Post Intervention; PT = Post-test; RCT = Randomized controlled trial; SCT = Social Cognitive Theory; TTM = Transtheoretical Model; VI-PA = vigorous intensity physical activity; WR = weight reduction
